# Supplementary material for: Human RNA Polymerase II Segregates from Genes and Nascent RNA and Transcribes in the Presence of DNA-Bound dCas9
Source: Int J Mol Sci. 2024 Aug 1;25(15):8411. doi: 10.3390/ijms25158411 (PMC11312690; doi:10.3390/ijms25158411)
Supplement: Supplementary file 1 [file ijms-25-08411-s001.zip › Pessoa_Carvalho_SupplementaryInfo_Proof.pdf]

# Human RNA polymerase II segregates from genes and nascent RNA and transcribes in the presence of DNA-bound dCas9

João Pessoa[1,2,*] and Célia Carvalho[1,3]

[1] *Instituto de Medicina Molecular João Lobo Antunes, Faculdade de Medicina da Universidade de Lisboa, 1649-028 Lisboa, Portugal*

[2] *Department of Medical Sciences and Institute of Biomedicine - iBiMED, University of Aveiro, 3810-193 Aveiro, Portugal*

[3] Email address: celiacarv@edu.ulisboa.pt

* Correspondence: joao.pessoa@ua.pt

# Supplementary Information

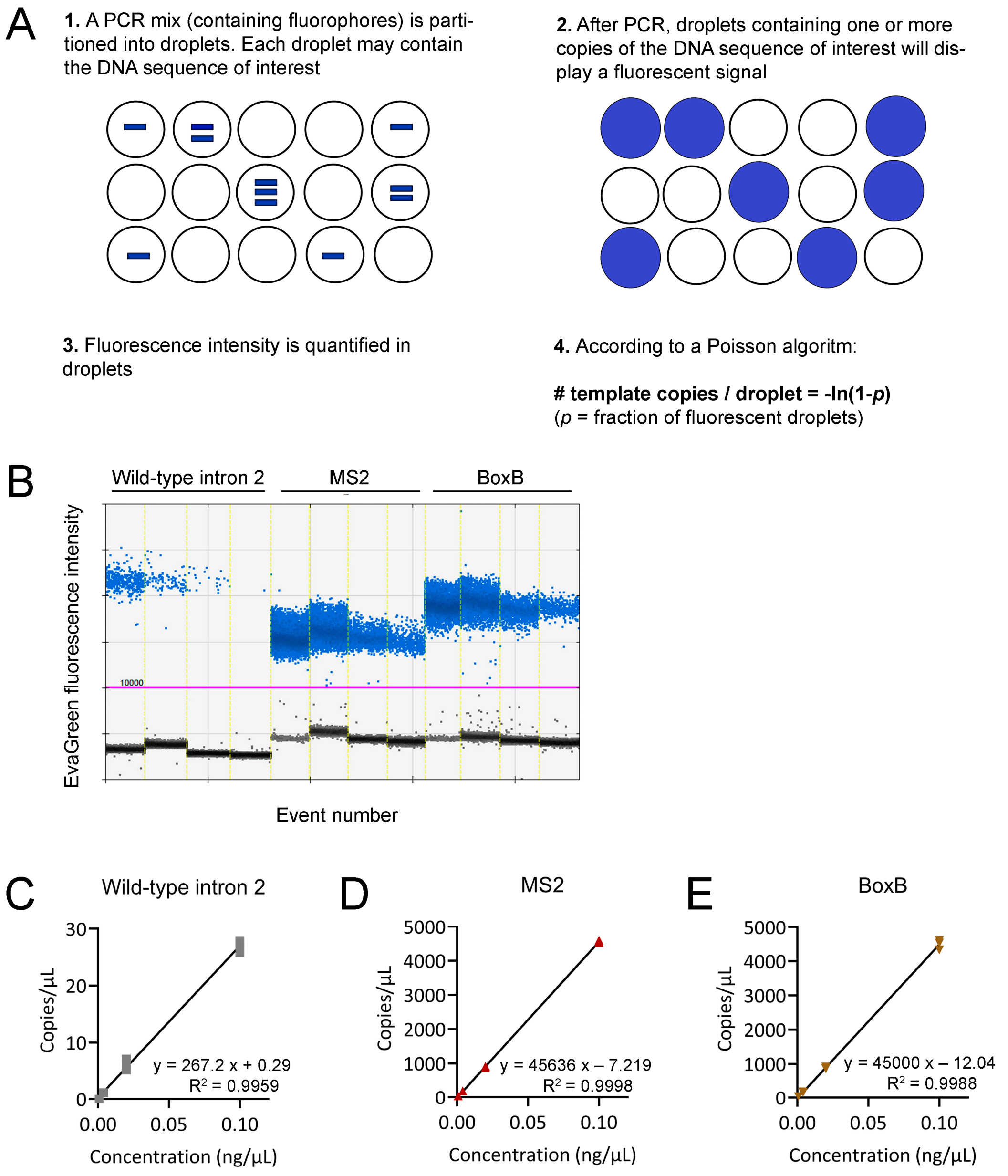


**Figure S1. Dilution effect in β-globin gene copy number determination by droplet digital PCR (ddPCR).** (A) Description of the methodology, in which template DNA and fluorescent droplets are represented in blue. (B-E) Linearity of β-globin gene copy number through serial dilutions. (B) Representative ddPCR raw data after amplification with primers specific for the wild-type intron 2 sequence of endogenous β-globin gene, or for the MS2 or BoxB regions of recombinant construct, in serial dilutions of genomic DNA from the multiple copy monoclonal cell line (see Figure 1F). (C-E) Graphical representation of concentration (copies/µL) obtained by ddPCR for each dilution, using each primer pair: wild-type intron 2, MS2, and BoxB, respectively. Linear regression equations and correlation coefficients are depicted, showing a notorious linearity with dilution. The calculation of the ratio of slopes of the transgene to endogenous control, times 2, reveals a copy number per genome of 339.2 ± 3.4 transgenes.

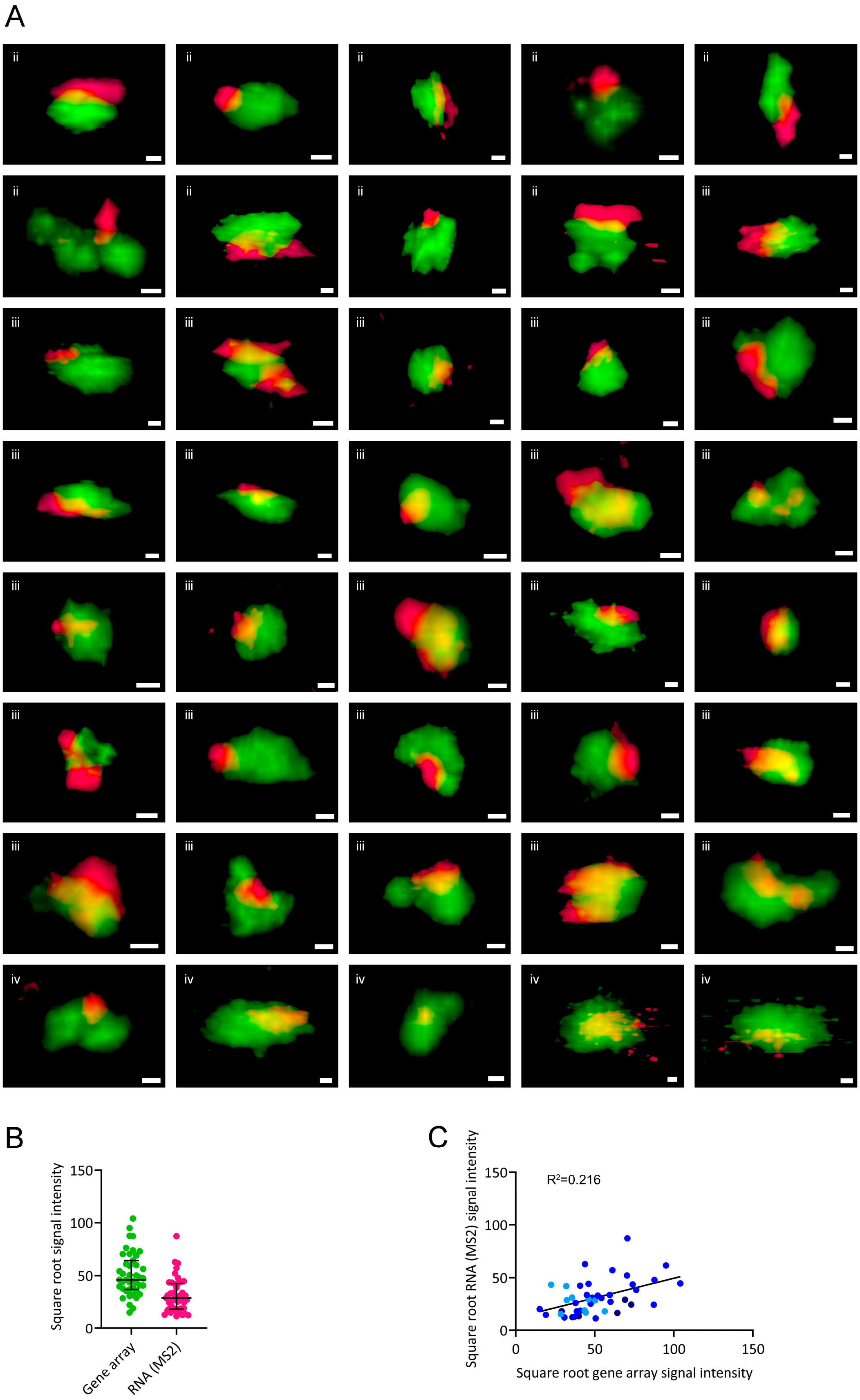
A
ii
iii
iv
B
Square root signal intensity
150
100
50
0
Gene array
RNA (MS2)
C
Square root RNA (MS2) signal intensity
R²=0.216
150
100
50
0
0
50
100
150
Square root gene array signal intensity

**Figure S2. 3D reconstructions of the interactions between a tandem array of ~335 recombinant human β-globin gene copies and their MS2-labelled nascent RNA, in U2OS cells.** (A) The gene array and its nascent RNA are represented in green and magenta, respectively. Their overlap extent is categorized as minor (ii), major (iii), and full (iv) overlap. Scale bars correspond to 0.3 μm. These reconstructions, in conjunction with those displayed in Figure 2B, constitute the full dataset analyzed in Figure 2. (B) Quantification of signal intensity of the gene array and MS2-labeled nascent RNA signals in the dataset. (C) Spearman correlation analysis between the signal intensities of the gene array and MS2-labeled nascent RNA. The coefficient of determination is indicated. A linear regression curve was fitted to the data points.

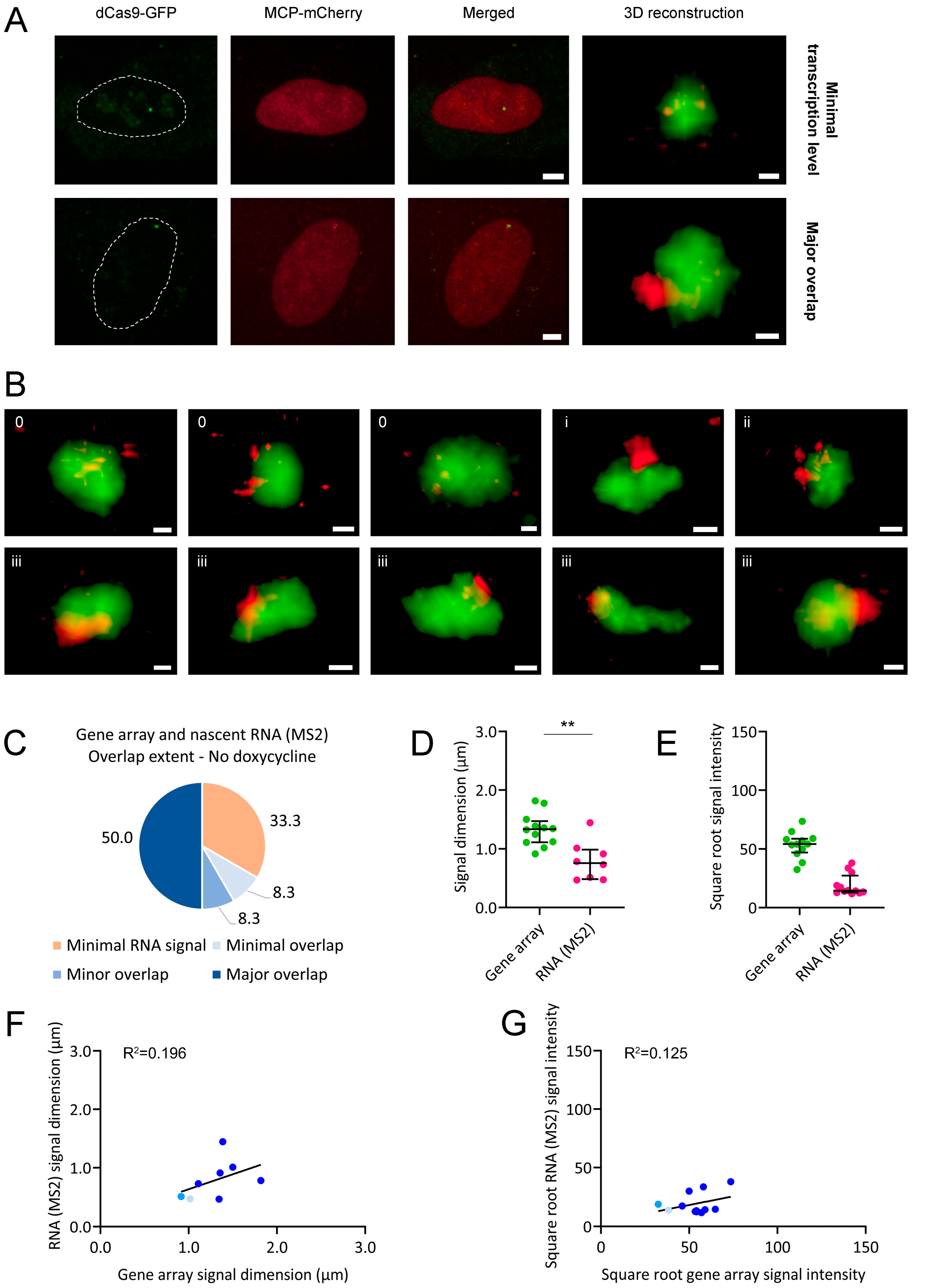


**Figure S3. Visualization of an array of ~335 recombinant human β-globin genes and their MS2-labelled nascent RNA, in the absence of transcription induction, in living U2OS cells.** (A)

Representative examples of spinning-disk confocal micrographs and corresponding 3D reconstructions, showing the fluorescently labeled human β-globin gene array (green) and its MS2-labeled nascent RNA (magenta). The dashed line represents the nuclear membrane. Scale bars correspond to 5 μm and 0.3 μm in single-plan micrographs and 3D reconstructions, respectively. (B) Additional 3D reconstructions, which in conjunction with those shown in panel (A), constitute the full dataset analyzed in this figure. Their overlap extent is categorized as minimal (i), minor (ii), and major (iii) overlap. In 3D reconstructions labeled with "0", the levels of nascent RNA were insufficient to assess their overlap extent with the gene array. (C) Quantification of the overlap extent in the dataset, where the percentages of each extent type are indicated near the pie graph. The percentage of 3D reconstructions with a minimal RNA content is represented in beige. (D-E) Quantifications of the signal (D) length and (E) intensity of the gene array and MS2-labeled nascent RNA signals in the dataset. Statistical significance was assessed with the Mann-Whitney test (**: $p<0.005$). (F-G) Spearman correlation analyses between the signal (F) lengths and (G) intensities of the gene array and MS2-labeled nascent RNA. Signals with minimal RNA content were not included in panel (F). The coefficients of determination are indicated. Linear regression curves were fitted to the data points. Data points in panels (F-G) are colored according to their overlap extent in panel (C). Results from panels (C) to (G) were compiled from 12 3D reconstructions of 12 single cells from one single experiment.

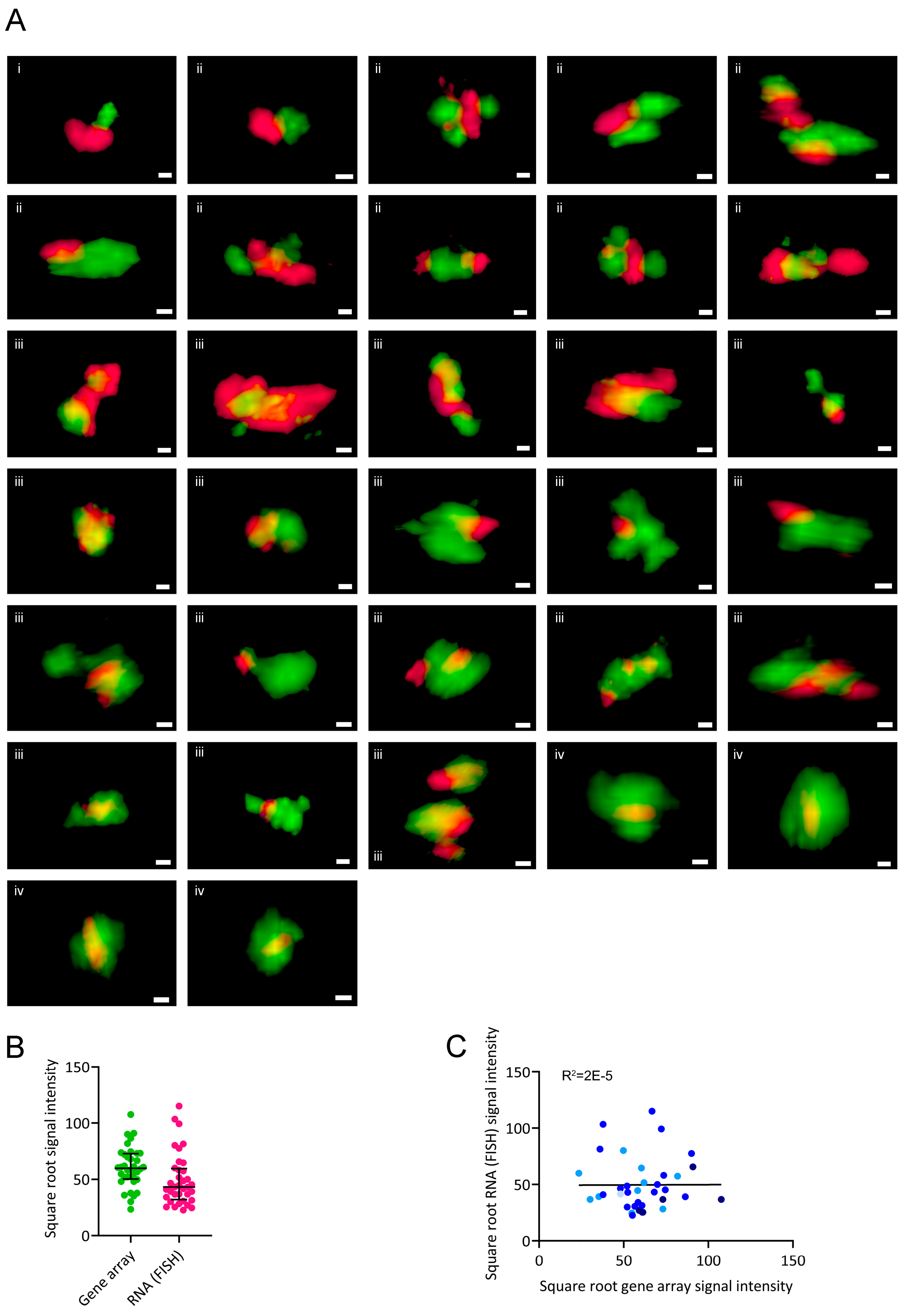

A
i
ii
iii
iv
B
Square root signal intensity
150
100
50
0
Gene array
RNA (FISH)
C
R²=2E-5
Square root RNA (FISH) signal intensity
Square root gene array signal intensity
0
50
100
150

**Figure S4. 3D reconstructions of the interactions between a tandem array of ~335 recombinant human β-globin gene copies and their FISH-labelled nascent RNA, in U2OS cells.** (A) The gene array and its nascent RNA are represented in green and magenta, respectively. Their overlap extent is categorized as minimal (i), minor (ii), major (iii), and full (iv) overlap. Scale bars correspond to 0.3 μm. These reconstructions, in conjunction with those displayed in Figure 3B, constitute the full dataset analyzed in Figure 3. (B) Quantification of signal intensity of the gene array and FISH-labeled nascent RNA signals in the dataset. (C) Spearman correlation analysis between the signal intensities of the gene array and FISH-labeled nascent RNA. The coefficient of determination is indicated. A linear regression curve was fitted to the data points.

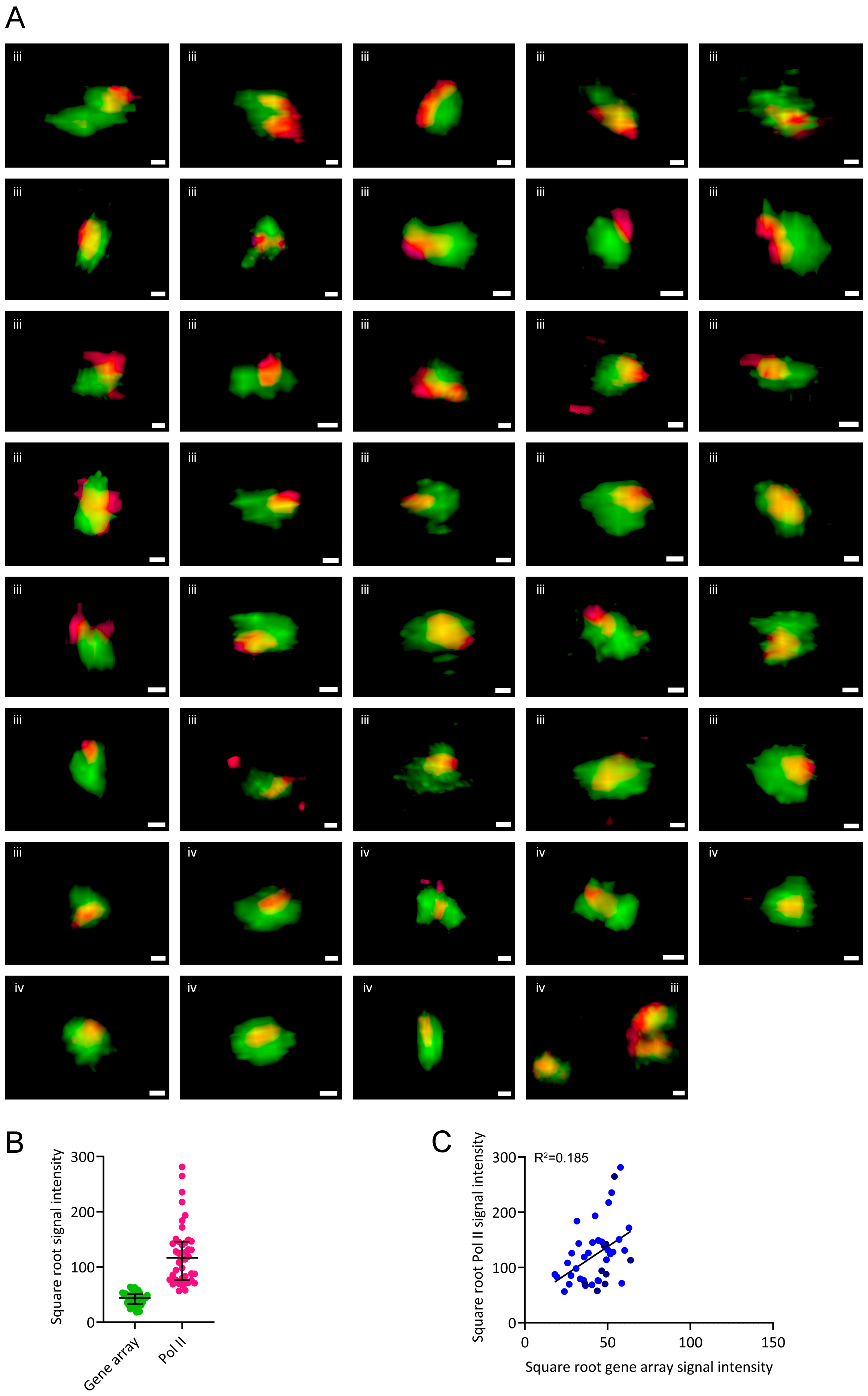
A
iii
iv
B
Square root signal intensity
300
200
100
0
Gene array
Pol II
C
R²=0.185
Square root Pol II signal intensity
Square root gene array signal intensity
0
50
100
150

**Figure S5. 3D reconstructions of the interactions between a tandem array of ~335 recombinant human β-globin gene copies and their transcribing Pol II, in U2OS cells.** (A) The gene array and its associated Pol II are represented in green and magenta, respectively. Their overlap extent is categorized as major (iii), and full (iv) overlap. Scale bars correspond to 0.3 μm. These reconstructions, in conjunction with those displayed in Figure 4B, constitute the full dataset analyzed in Figure 4. (B) Quantification of signal intensity of the gene array and transcribing Pol II signals in the dataset. (C) Spearman correlation analysis between the signal intensities of the gene array and Pol II. The coefficient of determination is indicated. A linear regression curve was fitted to the data points.

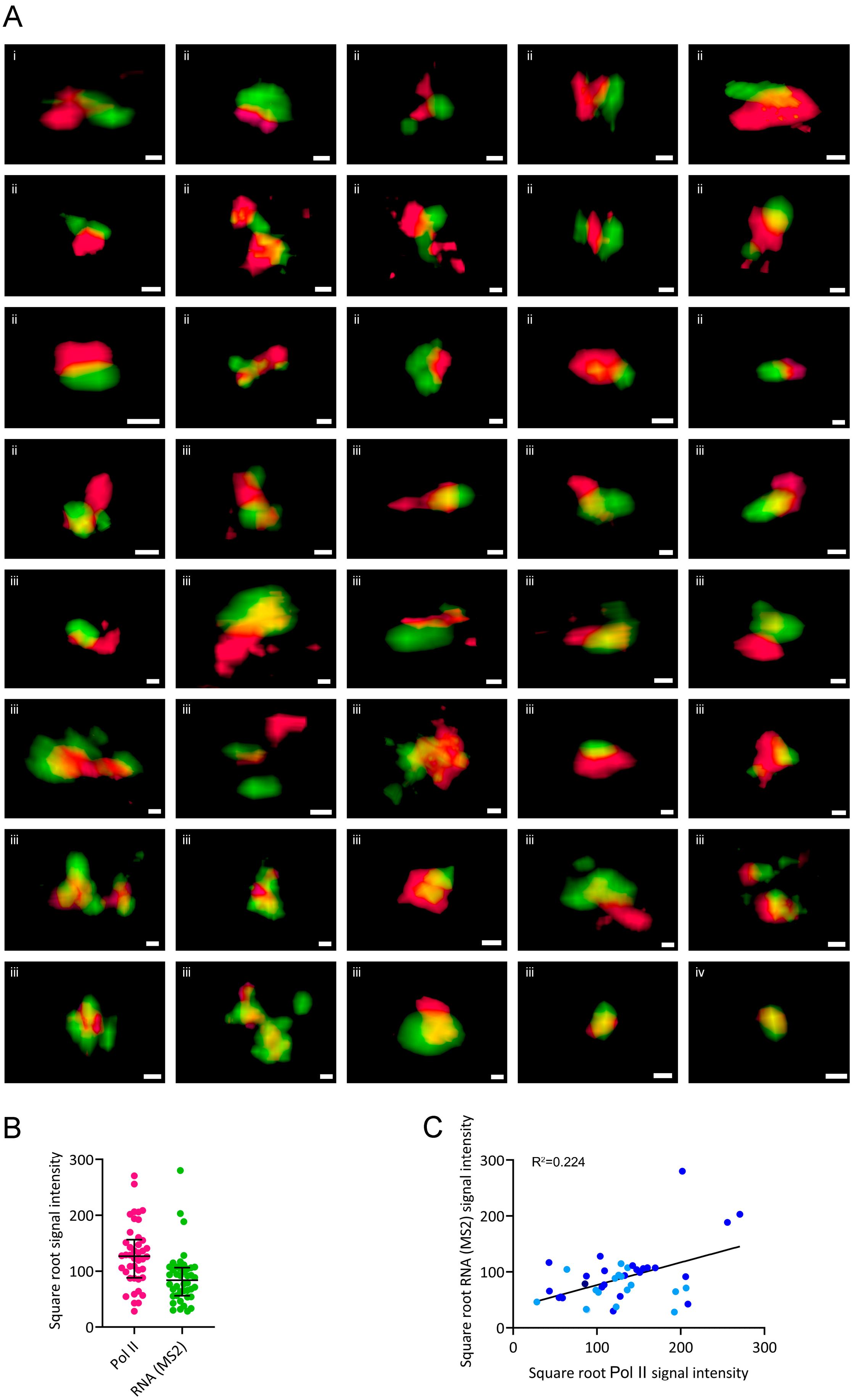
A
i
ii
iii
iv
B
Square root signal intensity
300
200
100
0
Pol II
RNA (MS2)
C
R²=0.224
Square root RNA (MS2) signal intensity
Square root Pol II signal intensity
300
200
100
0
0
100
200
300

**Figure S6. 3D reconstructions of the interactions between Pol II and its MS2-labelled nascent RNA transcribed from a tandem array of ~335 recombinant human β-globin gene copies, in U2OS cells.** (A) Pol II and its nascent RNA are represented in magenta and green, respectively. Their overlap extent is categorized as minimal (i), minor (ii), major (iii), and full (iv) overlap. Scale bars correspond to 0.3 μm. These reconstructions, in conjunction with those displayed in Figure 5B, constitute the full dataset analyzed in Figure 5. (B) Quantification of signal intensity of Pol II and MS2-labeled nascent RNA signals in the dataset. (C) Spearman correlation analysis between the signal intensities of Pol II and MS2-labeled nascent RNA. The coefficient of determination is indicated. A linear regression curve was fitted to the data points.

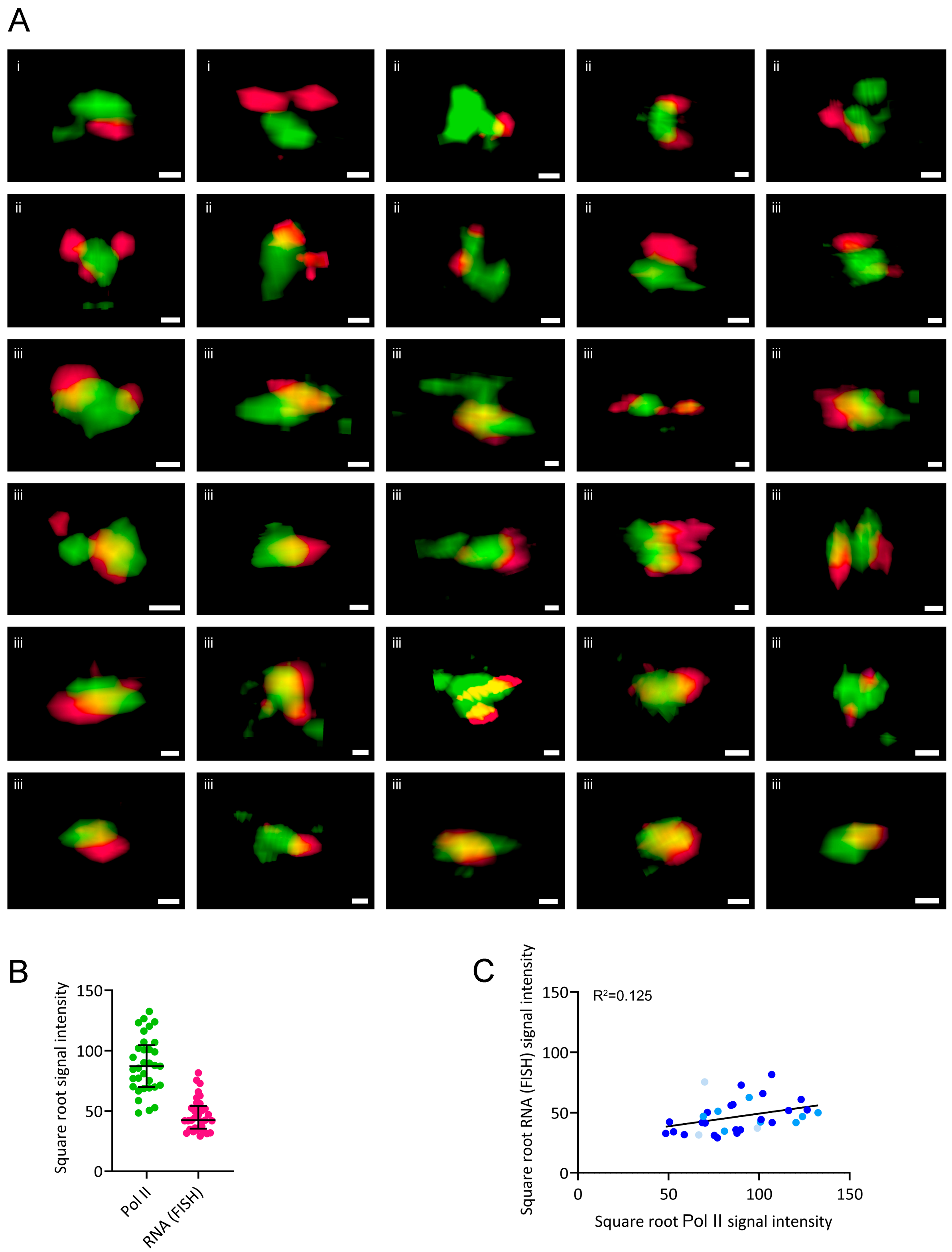


**Figure S7. 3D reconstructions of the interactions between Pol II and its FISH-labelled nascent RNA transcribed from a tandem array of ~335 recombinant human β-globin gene copies, in U2OS cells.** (A) Pol II and its nascent RNA are represented in green and magenta, respectively. Their overlap extent is categorized as minimal (i), minor (ii), and major (iii) overlap. Scale bars

correspond to 0.3 μm. These reconstructions, in conjunction with those displayed in Figure 6B, constitute the full dataset analyzed in Figure 6. (B) Quantification of signal intensity of Pol II and FISH-labeled nascent RNA signals in the dataset. (C) Spearman correlation analysis between the signal intensities of Pol II and FISH-labeled nascent RNA. The coefficient of determination is indicated. A linear regression curve was fitted to the data points.

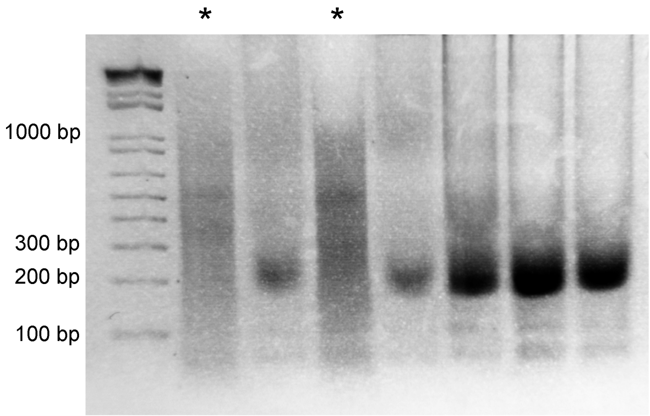


**Figure S8. Estimation of the size of DNA fragments used in chromatin immunoprecipitation.** 2% agarose gel analysis of samples that were not sonicated (indicated with asterisks) shows a smear spanning a wide kilobase pair range. All other samples, which were sonicated, reveal a broad, yet clearly defined band at about 200 bp.

**Video S1. Fluorescence recovery after photobleaching of dCas9-GFP bound to exon 2 of a recombinant human β-globin gene, in an array of ~335 gene copies, in the absence of doxycycline.** Each time point corresponds to 15 seconds.

**Video S2. Fluorescence recovery after photobleaching of dCas9-GFP bound to exon 2 of a recombinant human β-globin gene, in an array of ~335 gene copies, in the presence of 1 μg/ml doxycycline.** Each time point corresponds to 15 seconds.

**Video S3. Fluorescence recovery after photobleaching of dCas9-GFP bound to the operator of a recombinant human β-globin gene, in an array of ~335 gene copies, in the presence of 1 μg/ml doxycycline.** Each time point corresponds to 15 seconds.

**Video S4. Fluorescence recovery after photobleaching of dCas9-GFP nonspecifically bound to a nucleolus.** Each time point corresponds to 2 seconds.

**Table S1: DNA oligonucleotides used in the cloning of guide RNA (gRNA)-expressing plasmids**

| Name | Targeted strand | Sequence |
|---|---|---|
| Telomeric repeats forward | Template | CTTCGTTAGGGTTAGGGTTAGGGTTA |
| Telomeric repeats reverse | Template | AAACTAACCCTAACCCTAACCCTAAC |
| Exon 1 forward | Template | CTTCGAAGGTGAACGTGGATGAAGT |
| Exon 1 reverse | Template | AAACACTTCATCCACGTTCACCTTC |
| Exon 2 forward | Template | CTTCGTATGGGCAACCCTAAGGTGA |
| Exon 2 reverse | Template | AAACTCACCTTAGGGTTGCCCATAC |
| Exon 3 forward | Template | CTTCGAGAATTCACCCCACCAGTGC |
| Exon 3 reverse | Template | AAACGCACTGGTGGGGTGAATTCTC |
| Operator forward | Coding | CTTCGTACGTTCTCTATCACTGATA |
| Operator reverse | Coding | AAACTATCAGTGATAGAGAACGTAC |
| Exon 1 forward | Coding | CTTCGGTAACGGCAGACTTCTCCTC |
| Exon 1 reverse | Coding | AAACGAGGAGAAGTCTGCCGTTACC |

**Table S2: Primers used for gRNA amplification and re-cloning into a lentiviral vector**

| Name | Sequence |
|---|---|
| Exon 1 template forward | CCTTGGAGAACCACCTTGTTGGGAAGGTGAACGTGGATGAAGT |
| Exon 2 template forward | CCTTGGAGAACCACCTTGTTGGTATGGGCAACCCTAAGGTGA |
| Exon 3 template forward | CCTTGGAGAACCACCTTGTTGGAGAATTCACCCCACCAGTGC |
| Exon 1 coding forward | CCTTGGAGAACCACCTTGTTGGGTAACGGCAGACTTCTCCTC |
| Reverse | GATCCTAGTACTCGAGAAAAAAAGCACCGACTCGGTG |

**Table S3: Primers used for recombinant β-globin copy number determination by droplet digital PCR**

| Name | Sequence |
|---|---|
| Wild-type intron 2 forward | GCTCACCTGGACAACCTCA |
| Wild-type intron 2 reverse | AACGATCCTGAGACTTCCACA |
| BoxB forward | GAGCATCTGGATCTTGGGTTTCTGAT |
| BoxB reverse | GGACAGATCCCCAAAGGACTC |
| MS2 forward | GCTCACCTGGACAACCTCA |
| MS2 reverse | TAGTTGGACAGATCGTCCCATAGACT |

**Table S4: Primers used for chromatin immunoprecipitation analysis by quantitative PCR**

| Name | Sequence |
|---|---|
| Promoter/exon 1 forward | CTCGTTTAGTGAACCGTCAGATCGCC |
| Promoter/exon 1 reverse | AGGGCCTCACCACCAACT |
| Exon 2 forward | CTGCTGGTGGTCTACCCTTG |
| Exon 2 reverse | AGCTTGTCACAGTGCAGCTC |
| Exon 3 forward | GCAACGTGCTGGTCTGTGTGCTG |
| Exon 3 reverse | GGCAGAATCCAGATGCTCAAGGC |
| Intergenic region forward | GGCTAATCCTCTATGGGAGTCTGTC |
| Intergenic region reverse | CCAGGTGCTCAAGGTCAACATC |

**Table S5: Primers and probes used for nascent RNA quantification by droplet digital reverse transcription PCR**

| Name | Sequence |
|---|---|
| Intron 1 – exon 2 forward | AGGCACTGACTCTCTCTGC |
| Intron 1 – exon 2 reverse | ACTTTCTTGCCATGAGCCTTC |
| Intron 1 – exon 2 (FAM-labeled probe) | TCCCACCCTTAGGCTGCTGGTG |
| GAPDH intron 2 – exon 3 forward | CATCCCTGTCCGGATGCTG |
| GAPDH intron 2 – exon 3 reverse | CTCGCTCCACCTGACTTCC |
| GAPDH intron 2 – exon 3 (HEX-labeled probe) | TGGCGGCCGCTCTACCGC |

Abbreviations used: GAPDH, glyceraldehyde 3-phosphate dehydrogenase; FAM, fluorescein; HEX, hexachloro-fluoresceine
